# Podocytes … What’s Under Yours? (Podocytes and Foot Processes and How They Change in Nephropathy)

**DOI:** 10.3389/fendo.2015.00009

**Published:** 2015-02-23

**Authors:** Chris R. Neal

**Affiliations:** ^1^Bristol Renal, University of Bristol, Bristol, UK

**Keywords:** podocyte foot processes, nephropathy, glomerular filtration barrier, subpodocyte space, podocyte cytoskeleton, actin cytoskeleton

## Abstract

Most of the described structures of podocytes in health and disease have been inferred from light and electron microscopic studies of rodent models. The variation in filtration barrier features is measured on micrographs, the aim being statistical significance. This is the technical campaign waged against kidney disease but this approach can be misleading. The signaling cascades and connectivity of the podocyte and foot processes (FPs) are inferred from *in vitro* studies that at best blurr the reality of the *in vivo* state. This review will outline actin signaling connectivity and the key differences in the structural and functional domains squeezed into the FPs and the relationship of these domains to other parts of the podocyte. It covers the changes in podocytes during nephropathy concentrating on FP and finally proposes an alternative interpretation of FP ultrastructure derived from articles published over the last 60 years.

## Introduction

**Figure 1:**
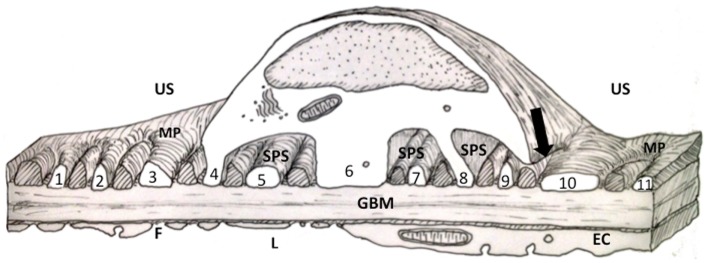
**Diagram of a thick section through the cell body of a podocyte showing foot processes (FPs)**. FPs arising from the two major processes (MP) in the diagram are labeled with numbers. Other FPs (cross hatched) interdigitating with the numbered FPs would have emerged from the MP of a podocyte neighbor (for further discussion of this figure see the text). EC, endothelial cell; F, fenestration; GBM, glomerular basement membrane; MP, major process; L, capillary lumen; US, urinary space; SPS, subpodocyte space.

**Figure 2:**
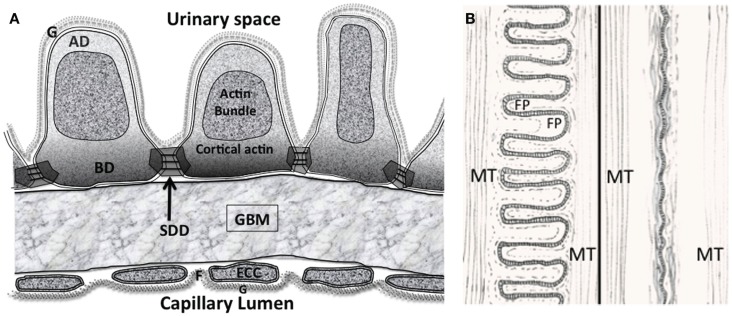
**(A)** The glomerular filtration barrier (GFB) with the disposition of membrane associated domains in foot processes (FPs). Diagram shows three FPs with apical domain (AD), basal domain (BD), and slit diaphragm domains (SDD) outlined. The apical actin bundles are shown, other actin networks cortical actin (sub-plasmalemmal actin) surrounding the actin bundle are attached to the various membrane domains. The cortical actin meshwork in BD and SDD is thicker than AD. ECC, endothelial cell cytoplasm; F, fenestration; G, glycocalyx. **(B)** Interdigitating foot process (FP) pattern in health (left) and after effacement in disease (right). The dashed line in FP marks the boundary of the actin bundles. MT – microtubules in the major processes. Actin bundles and cortical actin either reduce to a diffuse band of actin after effacement or disperse with effacement.

The podocyte has an intrinsic part to play in forming and maintaining the glomerular filtration barrier (GFB), but the relevance of the various structural components of the GFB in disease is complex (Figure 1). For instance, the glomerular basement membrane (GBM) not only serves as a barrier to protein *in vivo* but also requires the slit diaphragm (SD) to prevent albumin passage from the capillary lumen into urinary space (1) (Figure 2A). In addition to SDs, the glycocalyx overlying the endothelial cells restricts macromolecular passage and ensures that plasma albumin is largely excluded from the GFB (2). However, it is mutations of SD proteins that are strongly linked to the occurrence of proteinuria (3–5), on this basis SDs are assumed to be the weakest point. The GBM after development is maintained by the podocyte with dysfunction leading to GBM disruption. However, changes in the underlying GBM can lead to podocyte dysfunction, which comes first in disease is unclear.

This review will outline the current view on podocyte structure focusing on the podocyte foot processes (FPs) with an overview of associated FP actin signaling and connectivity. Additionally, regulation and instigation of structural changes associated with disease states will be outlined. Finally, alternative interpretations of FP structure and FP changes will be advanced based on the presence of a subpodocyte space (SPS) (Figure 1).

## “Normal” Podocyte Structure

Podocyte ultrastructure has been investigated for over half a century (6) with the GFB model being developed continuously (7). The urinary space of the glomerulus has been described as free to the movement of fluid and small solutes with the GFB being the single fluid resistance between the blood and urinary spaces (8).

A large number of reviews and articles published in the scientific literature cover the current description of normal podocyte structure. Briefly, the cell body has many major processes (MP) attached to it, primary, then secondary, and some tertiary MPs branching from the cell body. FPs emerge at right angles to the MPs and make contact with the underlying GBM (9) (Figures 1 and 2A).

Foot processes from neighboring podocytes interdigitate with each other forming a SD with its heavy investment of specific proteins and structures (10). The importance of podocyte SD structure is highlighted by exact homologs in insect nephrocyte SDs employing similar conserved proteins for hemolymph filtration (11). The highly conserved structure and function between insects and vertebrates illustrates that nephrocytes and podocytes are from a very old cell lineage having evolved on filtration barriers *at least* since the vertebrate/insect last common ancestor over half a billion years ago (11).

## Podocyte Cytoskeleton

Aside from the longitudinally oriented actin bundles in the central apical part of FPs, there are three membrane associated domains (Figure 2A). A sub-plasmalemmal network of cortical branched actin filaments connects the SD domains (SDD), the basal domain (BD), and the apical domain (AD) to the actin bundle (12, 13). At the end of FP adjoining major processes (MPs), actin bundles connect to MP intermediate filaments and microtubules running back to the central cell body (14–17) (Figures 2A,B). The complete FP cytoskeleton is reported as having the components necessary to oppose the forces of a high pressure distensible glomerular capillary wall (18–20). The FP actin network should not be confused with MP and cell body actin stress fibers observed in both *in vitro* and *in vivo* podocytes (21).

Podocyte actin filaments organized into stress fibers are in contrast to the FP actin network attached to the cell membrane thru focal adhesions (18). They regulate cell motility and any sustained cell body contractions, the structure is similar to that of sarcomeres in myocytes with myosin filaments, α-actinin, and regulators like tropomyosin (22). Tropomyosin is only found in the cell body of podocytes the equivalent regulator in FP actin networks being synaptopodin (23).

Small GTPase Rho associated protein kinase ROCK as well as intracellular calcium levels are the main upstream regulators of myosin activity. Rho and calcium pathways regulate actin stress fiber dynamics of the cell body (24) as well as the cortical actin and actin bundles of FP but with different regulators (see above).

Microtubules and intermediate filaments are not part of FP structure. Differentiated podocytes *in vivo* have a mesenchymal intermediate filament pattern with the cell body and MPs expressing vimentin and desmin (17, 25, 26) and intermediate filament associated proteins plectin and p250 protein (27, 28).

### Endocytosis and exocytosis in foot processes

While endothelial cells and podocytes produce the GBM in development, the fully differentiated podocyte assembles and secretes matrix components into the GBM via the BD. This maintains the GBM meshwork of collagen IV, laminin, fibronectin, entactin, agrin, and perlecan (29). Cargos from production sites closer to the cell body (Golgi apparatus, rough ER) necessarily require transport to FPs via MP microtubules (see below). Endocytotic transmembrane proteins exist along the BD membrane (e.g., megalin) and motor proteins that bind membranes to actin such as myo1e are recruited in clathrin endocytosis (30). Ultimately, these cargoes will require transport to the cell body.

### Major process microtubular transport

The tubulin subunits form a stiff 24 nm-thick tubular structure along the length of MPs and connect the cell body with FP actin networks. The minus-ends of microtubules can be located in the cell body microtubule organizing center with the plus-end (fast growing) at the cell periphery, resulting in a neuronal “plus-end-distal” orientation (31). However, podocyte microtubules are orientated both ways, allowing transport and elongation in either direction along MPs (16, 32). Central to distal cargo transport was shown with movements of virus (33) and also vesicular cargos were moved from a Golgi apparatus under the control of rab8 (34), another small GTPase.

Evidence of distal to central movements has been seen with Wilms tumor 1 interacting protein (WT1P) an actin associated protein (AAP) in FP, which translocates from the SDD to the nucleus via dynein-microtubule transporters (35) [Stress signaling initiates loss of WTIP from SDD and suggests a mechanism that transmits changes in podocyte morphology to the nucleus (35)]. The genes coding for Nephrin (SDD) and podocalyxin (AD) are dramatically downregulated in mice with decreased levels of WT1 gene expression (36).

The CHO1/MKLP1 (kinesin superfamily motor protein) is responsible for elongation of minus-end-distal microtubules in podocyte processes (16). Microtubular associated protein 4 (MAP4) could be another microtubule forming molecule in podocytes (37) with phosphorylation slowing microtubule assembly along the process.

### Foot process actin connectivity

**Figure 3:**
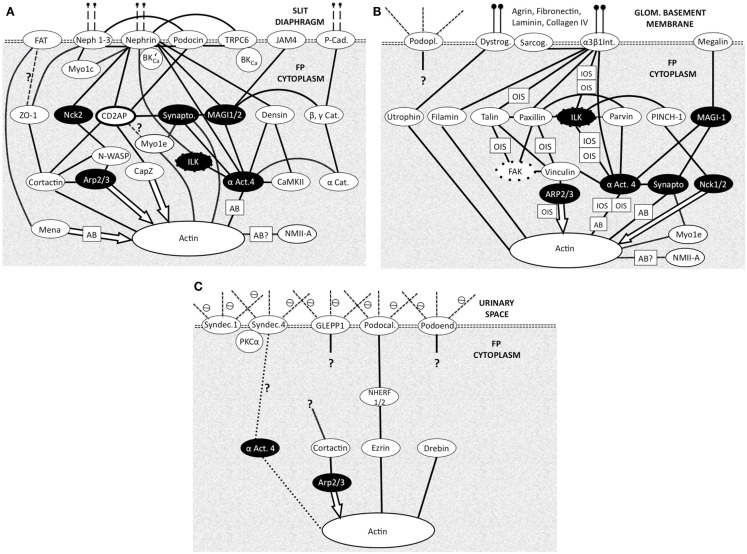
**Foot process membrane and associated domains with membrane proteins at the top then adapter and effector proteins leading to actin**. Each domain is connected to the underlying cortical actin network and to the other domains. (N.B. Not all protein interactions are shown) all are bidirectional biochemical interactions except effector molecule interactions (arrows). Question marks show uncertainty. White text on black shows common adapter/effector molecules across the three domains. Most actin interactions will be with local cortical actin with some potential longer range actin bundle interactions (AB) Figure adapted from Refs. (13, 38, 39). **(A)** Slit diaphragm domain (SDD). Vertical dashed lines at the top indicate connectivity across the SD to another FP. Cat, catenin; αAct4-α, actinin4; Cad, cadherin; Synapto, synaptopodin; BK’_Ca_ – BK_Ca_, splice variant, which binds Nephrin; Myo1c, myosin 1c; Myo1e, myosin 1e; NMIIA, non-muscle myosin II A; *Actin action*: *Mena, organizes long actin into bundles inhibits capping. Cortactin, promotes branching. Arp 2/3, branching and actin nucleation. CapZ, actin end capping. α Act.4, actin bundler and anchorer. Synaptopodin, actin bundler*. **(B)** Basal domain (BD). Vertical lines at the top indicate attachment to GBM components. Dystrog, dystroglycan; Sarcog, sarcoglycan; α3 β1 Int, α3 β1 integrin; αAct4, α actinin 4; Podopl, podoplanin (gp38); OIS, outside-in signaling (integrins regulate actin dynamics); IOS, inside-out signaling (actin regulates integrin adhesiveness); NMIIA, non-muscle myosin type IIA. *Actin action*: *Filamin, binds 2 actins at large angles (to make networks). Utropin, binds along actin length. NMIIA, motor protein binding 2 actins. Myo1e, motor protein binding membrane to actin. Nck1/2, actin polymerization*. **(C)** Apical Domain (AD). Dashed lines at the top indicate proteoglycan part of the membrane proteins in urinary space. Syndec, syndecan; αAct4, -α actinin 4; ⊖, negative charge on proteoglycans (glycocalyx) *Actin action*: *Ezrin, actin binder. Drebin, actin binder*.

An ever increasing number of actin regulators adapters and associated proteins are being discovered around the cortical actin of the FP membrane domains and around the apical actin bundle (see Box 1). The distribution of FP proteins around the different domains is crucial with different biochemistries setting different signaling. Some of the major interactions with actin involved in FP structural change are described below and in Figure 3 [for a fuller description of the protein complexes see references (13, 38, 39)]. The type of actin interaction by the final signal molecule in the pathway is also described in Figure 3.

Box 1 **Podocyte actin associated proteins.**

The cytoskeletal research impetus has been on the actin cytoskeleton since the only identified mutants in patients have all been based around this part of the cytoskeleton and no mutations have been identified so far in microtubule or intermediate filament associated genes (40). Any disruption of microtubule function with colchicine or vinblastine did not appear to alter FP dynamics in the short term (41) (although the trafficking of cargoes between the cell body and the GBM should result in GBM disruption in the longer term). Any proteinuric kidney disease that can not be traced to podocytic actin filament disruption is traceable to slit diaphragm change (42).

Podocyte actin research momentum has revealed a huge number of actin associated proteins. A list compiled by Faul showed that 94 actin associated proteins (AAPs) have been discovered in cultured mammalian podocytes up to 2013. This can be narrowed down to a list of 26 that have mutations associated with human disease or genetic modification resulting in experimental proteinuria (43). How these AAPs fully interact with the individual podocyte actin domains and networks especially in the FPs remains to be determined.

The list is ever growing: two further podocyte cytoskeletal proteins have been found upregulated in the FPs of puromycin aminonucleoside nephropathy (PAN) rats and also human disease (44). Survivin (Birc5 gene) colocalizes with synaptopodin and is an actin binder, survivin knockdown with siRNA rearranges the actin cytoskeleton (45). Another potential is Arc/Arg3.1 (Activity regulated cytoskeleton associated protein), which acts as a crucial mediator for actin polymerization in other cells. Its possible Arc is associated with podocyte endocytotic control, weirdly, the gene was upregulated but not the protein in PAN rats (44, 46).

### Slit diaphragm domain actin coupling in FPs

In the SD region cortical (sub-plasmalemmal), actin is connected to the molecules of the SD complex in the membrane (SDD Figure 2A). ZO-1 (47), catenins (48), CD2AP (49), and podocin (50) and the like serve as adapter molecules between the slit membrane molecules nephrin, Neph, and P-cadherin and actin microfilaments. α-actinin 4 acts as a particularly busy signaling node (Figure 3A), it is notably abundant in podocyte FPs and colocalizes with all actin associated with the FPs (51). Arp2/3 also represents a prominent signaling node. Other classical actin binding proteins function in different modes – such as the non-muscle myosin Myo1e, which signals between synaptopodin and actin and Myo1c interacts directly with nephrin and Neph1 ensuring insertion in the SDD (38, 39).

### Apical domain actin coupling in FPs

In the AD (Figure 3C), Syndecans I and IV, podocalyxin GLEPP1, and podoendin are major contributors to the surface negative charge on the apical surface (52–54), which is said to repel proteins and act as a spacer molecule preventing FPs from getting too close (55). Compared to the SDD, less is known about actin signaling in the AD. Podocalyxin is joined to the sub-plasmalemmal actin system via NHERF2 and ezrin (56) podocalyxin complex disruption results in changes in FP structure (57). Podocalyxin null mice fail to form FPs (58); even though these changes are in an apical position away from the SDD and BD.

### Basal domain actin coupling in FPs

A thicker meshwork of actin is found in the BD facing the GBM and also the lateral SDD actin network. In the BD of FPs (Figure 3B) dystroglycan connects actin to agrin in the GBM independently of α-actinin 4 but a large part of the signaling again centers around α-actinin 4. Megalin, a transmembrane endocytotic receptor glycoprotein links to actin via α-actinin 4 and synaptopodin (59, 60) and the integrin complex (5) acts through α actinin 4.

Signaling from the GBM to the integrins can regulate actin dynamics in outside-in signaling (OIS; Figure 3B) but inside-out signaling (IOS; Figure 3B) also occurs where actin signaling to the integrin complex alters integrin adhesiveness. Inside-out IOS signaling can also signal via arp2/3 and vinculin (Figure 3B) (61).

### Prominent FP Signaling Nodes

Common signaling components of the three domains are highlighted in black throughout Figure 3. These are seen as prominent nodes and important in the different signaling pathways of the three domains. α-actinin 4, Arp2/3, integrin-linked kinase (ILK), and synaptopodin are highlighted below.

Irrespective of membrane domain α-actinin 4 represents a prominent signaling node in the three domains but with noticeably different connectivity to the cortical actin and actin bundles of the FP. The network associations in Figure 3 show α-actinin 4 to be closely associated with the cortical actin network and also active in the actin bundles of FP. Immuno-staining of α-actinin 4 shows location in only the actin bundles in rats (12), whereas in human podocytes its confined to SDD or BD and not in an apical actin bundle position (62). The close association with other proteins in complexes could cloak the α-actinin 4 antigenic site as it interacts directly with integrins in BD (Figure 3B) and forms a complex with nephrin and ILK in SDD (63) (Figure 3A). This highlights caution in interpretation of immuno-staining results.

Arp2/3 is in a separate signaling pathway to α-actinin 4 in SDD but both interact with vinculin in BD (Figure 3B). Since Arp2/3 interaction with actin is as an effector then only outside-in signaling (OIS) is possible. Its associations in the AD are still being defined (Figure 3C).

Integrin-linked kinase (Figures 3A,B) seems to function as an adaptor that biochemically and functionally connects the BD and SDD complexes (13, 61). In the SDD, ILK physically interacts with nephrin to form a ternary complex, and α-actinin-4 in ILK/nephrin complex formation (63) in BD one of its functions is in relaying signals from integrins to actin (61) (Figure 3B).

Synaptopodin can be described as a scaffolding protein connecting the signaling complexes of the SDD and the BD by virtue of binding to α-actinin and the actin cytoskeleton (13). Small GTPases like RhoA act as regulators of actin networks and synaptopodin is involved in the promotion of RhoA-mediated actin fibril formation at the SDD or BD next to the GBM. However, in AD podocalyxin can activate RhoA (64).

## Podocyte Changes in Disease

Disruptions in podocyte biochemistry will alter podocyte structure in disease. The structural changes will be outlined in this next section and examples given of the underlying signaling.

### Reduction in podocyte number

A reduction in podocyte number per glomerulus is a feature of FSGS (65, 66). Wharram showed a direct relationship between reduction in the podocyte population and extent of glomerulosclerosis in rats (67), podocyte depletion marks an inability of the glomerulus to replace podocytes. Detachment of podocytes due to disruption of cell adhesion proteins in the BD (68) allows recovery of them as viable cells from urine (69). Apoptosis regulated through cell cycle regulatory proteins (cyclin) and/or caspase routes can be an early feature in some disease as in type 2 diabetic kidney disease (70).

Podocyte loss is prevalent in type 2 diabetes (with microalbuminuria) the lower density of podocytes per glomerulus was the strongest predictor of renal disease progression (71). Again, podocyte loss in type 2 diabetic nephropathy scaled with the progression of the disease (72). However, in type 1 diabetic kidney disease the frequency of all glomerular cells varied with age in one study but was *not* reduced in the diabetic compared to control. The glomerulus appeared to regulate its architecture to maintain a constant podocyte density (73, 74). Although the second study did show a correlation between podocyte loss and proteinuria it was uncertain whether it was cause or effect of the nephropathy (74).

### Podocyte division

How do neighboring podocytes fill in the gaps where podocytes are lost? Podocytes show no evidence of division *in vivo* (75), so how mature podocytes regenerate is unclear. Replacements from bone marrow have been demonstrated in mice (76, 77) other possibilities include the parietal cells of the Bowman’s capsule, which can migrate from the urinary pole to the vascular stalk and there differentiate into new podocytes (78–80). Using serial multiphoton imaging of podocin-confetti mice in a renal fibrosis model the appearance of a new podocyte was noted within 24 h (81). Thus, some proportion of the podocyte population or its precursors has a highly dynamic, motile, and migratory phenotype, which must involve actin stress fibers.

### Podocyte hypertrophy

Cell expansion is one possibility for filling the podocyte gaps in disease, aging rat podocytes have been shown to increase in size up to twofold. This starts as a non-stressed hypertrophy and progresses to a severe hypertrophy with a reduction in (functional) SD proteins, fattening of FPs and increased proteinuria. Loss of SDs mark a reduction in filtration capacity; however, the resultant GFB still has a podocyte cover. The final stage involves more podocyte loss and glomerulosclerosis (82). Podocyte hypertrophy occurs with glomerular capillary hypertension ultimately leading to progressive glomerulosclerosis (83).

The controllers and regulators of hypertrophy are not completely resolved. A neuronal protein ubiquitin C-terminal hydrolase L1 (UCH-L1) appears to induce podocyte hypertrophy in Membranous Glomerular Nephritis (MGN) by increasing the total protein content by promoting cytoplasmic accumulation of proteins such as Cdk inhibitors (p27^Kip1^) (84). Modification of both UCH-L1 activity and levels could reduce podocyte hypertrophy therapeutically in MGN.

The cyclin-dependent kinase inhibitor p27^Kip1^ is a major regulator of the podocyte hypertrophic response to hyperglycemia *in vitro* (85) and *in vivo* in a mouse type 2 diabetes model (86) and levels of p27^Kip1^ radically increase in experimental nephritis (87). However, different Cdk inhibitors appear important in podocytes *in vitro*, (88) compared to *in vivo*.

Another regulator of hypertrophy is GLUT4 (an insulin downstream effector), deficiency in podocytes results in fewer hypertrophic cells. GLUT4 also protects mice from the development of diabetic nephropathy (no proteinuria). There is a possibility that podocyte hypertrophy concomitant with podocyte loss may be associated with a protective mechanism avoiding proteinuria.

Genetic deletion of mTOR complex 1 (mTORC1) in mouse podocytes induced proteinuria and progressive glomerulosclerosis. However, increased mTOR activity accompanied human diabetic nephropathy, characterized by early glomerular hypertrophy and hyperfiltration. These results demonstrate the requirement for tightly balanced mTOR activity in podocyte homeostasis and suggest that mTOR inhibition can protect podocytes and prevent progressive diabetic nephropathy (89).

### FP effacement

The next response of FPs to disease or abnormal conditions is to retract toward the MP, any remaining FPs spread out and the normal interdigitating nature of the FPs gets simplified and smoothed into an undulation (Figure 2B). In a review on FP effacement, Kriz et al. pointed out that the process may be an adaptive and protective response in order to escape cell detachment or to cover bare areas where cells have been removed (90). This is in contrast to the old idea of damage and injury.

Effacement can be rapidly induced after only a few minutes of protamine sulfate, which reduces podocyte surface charge (41) with no observable change in SDs which apparently remain intact. α-actinin4 was found in effaced FP basal actin networks consistent with FP effacement being an adaptive change in cell shape, reinforcing the supportive role of podocytes (91).

### Actin network failure

Foot process effacement occurs with the disruption or dispersion of its actin networks. Rat glomerulopathy effacement occurs with buildup of a meshwork of intercrossing actin fibers on the FP BD adjacent to the GBM (91, 92). In contrast, the effacement of complement-mediated injury (93) or PAN (94) dissociated the basal actin cytoskeleton from matrix-attached integrins producing a transient dispersion of actin microfilament structure. How these structural changes relate or integrate with all the FP actin domains is unknown (12).

It seems there is a fine biochemical balancing act over activation or dispersion of actin basal networks, both resulting in FP effacement. PAN FP effacement was preceded by raised α-actinin 4 expression (91, 95) and transgenic overexpression of α-actinin 4 in mice yields an FSGS phenotype (96). However, mutations of ACTN4, reducing the levels of normal α-actinin 4, causes a late-onset FSGS (97) and the mutant crosslinkage with actin causes loss of nephrin from the SD. In signaling α-actinin-4 is heavily linked to both SDD (Nephrin, JAM4, p Cadherin; Figure 3A) and also the BD (via MAGI1 – megalin, α3 β1 integrin; Figure 3B) and possibly AD (Figure 3C).

The SDD, AD, and BDs of the FPs are physically linked to the FP cortical actin and central actin bundles, actin is the structural denominator in podocyte function and dysfunction (5, 98). Its clear that any interference with the three FP domains (BD, AD, SDD) and associated cortical actin then affects the FP actin bundles. These rearrange into either a dense network of short branching actin filaments (similar to cortical actin meshwork) or a dispersed network, both arrangements resulting in FP effacement and proteinuria.

### SD loss promoting disease

Reduction in cellular levels, mutation or dislocation of SD proteins leads to SD loss or disruption and FP effacement. SD protein mutations or closely associated FP protein mutations signpost FSGS. Nephrin, Podocin, CD2AP, PLCε1, and MYO1E (non-muscle myosin) are autosomal recessive; α-actinin-4, TRPC6, and INF2 (Inverted Formin 2) are autosomal dominant (99). The dislocation or loss of only one SD component is necessary and effacement can occur, in Passive Heymann Nephritis for instance, it is Nephrin alone that appears to dissociate from SDs (100).

### Small GTPase regulators of podocyte actin

The small GTPase group of proteins appears throughout in regulatory roles involving actin and podocyte shape change. Signals from the SD to actin and the microtubular system are critical in maintaining normal position, shape and functioning for the FPs. Critical regulators are RhoA, Rac1, and Cdc42. A GTPase activating protein binds CD2AP, which is present in the Nephrin complex (101), also nephrin can activate Rac 1 through a phosphoinositide pathway involving another nephrin binder Fyn (102) (not represented in Figure 3A).

Synaptopodin RhoA interaction seems to be critical for Actin fibre regulation (103). SD domain TRPC6 mediated calcium influx activates RhoA and inhibits podocyte motility. Either an increase or decrease in motility due to RhoA changes lead to FP effacement, too much or too little disrupts the barrier (104).

RhoA and Rac1 also appear in a similar balanced mechanistic model of podocyte disruption involving a circulating factor and podocyte protein expression. FSGS and DKD (diabetic kidney disease) have elevated levels of circulating soluble urokinase plasminogen activator receptor (suPAR) but the podocyte expression of acid sphingomyelinase-like phosphodiesterase 3b (SMPDL3b) is elevated only in DKD (105).

In FSGS, high SuPAR levels lead to Rac 1 activation and a migratory phenotype. In DKD, high suPAR levels competitively bind with high SMPDL3b, allowing RhoA activation and increased apoptosis (Table 1). Healthy podocytes with an absence of proteinuria mark a balance between the two.

**Table 1:** **The consequences of circulating levels (+, +++) of suPAR and podocyte expression levels (+, +++) of SMPDL3b on the activation (⇑⇑) or inactivation (⇓⇓) of integrins (αVβ_3_)**.

FSGS	Healthy	DKD
SMPDL3b+	SMPDL3b+	SMPDL3b+++
suPAR+++	suPAR+	suPAR+++
αVβ_3_ ⇑⇑	αVβ_3_ ⇔	αVβ_3_ ⇓⇓
RhoA ⇓Rac1 ⇑	RhoA ⇔Rac1 ⇔	RhoA ⇑Rac1 ⇓
Migration	–	Apoptosis
Proteinuria	–	Proteinuria

*High levels of SMPDL3b and suPAR competitively bind and inactivate αVβ_3._ Subsequently a different small GTPase. is activated leading to either a migratory or apoptotic phenotype. [Tabulated from Yoo et al. (105)]*.

## Problems with the Current Structural Model

The pathways of actin signaling highlighted here show that the different domains are complex highly modified evolved structures that have radically different signaling pathways in closely adjacent domains. From above it seems that disruption of often only a single element of the actin linked pathways in FP in any of the membrane associated domains leads to effacement. A more recent interpretation of podocyte structure raises the question of how basic FP signaling pathways are arranged in FP structure.

The view above simply assumes one type of FP and attached SD; however, a discovery 60 years ago, which was revisited in the 1980s and in 2000s challenges this view. The presence of a subpodocyte space (SPS) was highlighted early on in EM investigations of podocytes. Gautier et al. in 1951 (106) first highlighted the presence of “lacunaire peri capillaire” (pericapillary lacunae) under podocyte cell bodies. Elias studied the SPS in the 1980s (107) but again this was ignored in favor of a simple urinary space concept.

More recently, the SPS was entirely reconstructed from electron micrographs and showed that these little urinary spaces were restrictive to fluid outflow (108–110) (Figure 1). Evidence has since emerged from two labs, which found SPS *in vivo* to be a restrictive space, which trapped 10 kDa macromolecules (111, 112) but was not restrictive to smaller 450 Da molecules (111). Mathematical modeling of the SPS predicted that the fluid resistance of SPS plus GFB was 2.5 times the “ordinary” uncovered GFB and exquisitely sensitive to changes in the width of the exit regions leading from the SPS (arrow in Figure 1) (109). This is a problem for structure-based models predicting fluid permeability without accounting for an SPS contribution to the barrier (113).

In essence, an SPS is formed by the cell body and some supporting MPs of the podocyte covering up and roofing over more than 50% of the urinary side of the GFB (108–110). The covered GFB comprises underlapping MPs and attached FPs sitting on GBM with the usual fenestrated endothelium on the luminal side (Figure 1).

### FP redefinition

At first glance the GFB underlying the SPS seems to be identical to the GFB in non-SPS areas with SDs and domains formed between FPs of neighboring podocytes. However, not all FPs in SPS arise from MPs because some FPs emerge directly from the cell body (Figure 1. foot processes 4, 6 and 8). These processes still run parallel with other FPs and TEM image reconstruction shows them to be extended columns supporting the cell body (109).

**Figure 4:**
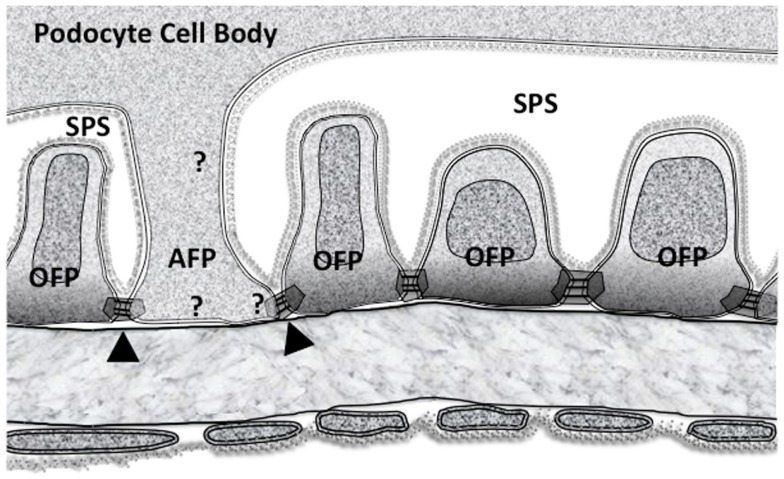
**The subpodocyte space (SPS) GFB showing the disposition of membrane domains in foot processes as in Figure 2**. Ordinary foot processes (OFP) have signaling networks that are well defined. The less frequent anchoring foot processes (AFP) could have some of the features of (OFP) but must have some differences based upon their altered response to increased perfusion pressure. Arrowheads mark the possible asymmetric slit diaphragm between OFP and AFP. Question marks show the possibility of differences in all three membrane associated domains.

These major FPs were originally referred to as anchoring processes (108, 109), here we call them anchoring foot processes (AFP) for clarity (Figure 4). The original name was applied when these processes were found to widen under transiently increased perfusion pressure (approximately 30 s), as if they were an adjustable anchor for the podocyte cell body on the GBM (108, 109). The smaller ordinary FPs (OFPs), which have all the cytoskeletal machinery for shape change did *not* widen, so AFPs appeared to be more sensitive to pressure than OFPs.

Looking at AFPs in more detail (Figure 4), there is an SD domain, a BD but an AD stretching up to the underside of the podocyte cell body (an SPS “ceiling” domain). If the OFPs and AFPs are considered separately, some initial questions arise:

### Contractile/cytoskeletal structures

1. Do AFPs have OFP type actin bundles, cortical actin, and membrane associated domains?
2. Do AFPs have cell centric actin stress fibers?
3. Since AFPs are a form of MP do they have microtubules?
4. Do AFPs have a combination of 1, 2, and 3.

### Signaling networks

1. What signaling networks and pathways are specific to AFPs compared to OFPs? Broadly, do the signaling pathways of “FPs” in Figure 3 occur in AFP?
2. With an increase in perfusion pressure how could the filtrate flow (through the SD) or mechanical stretch (of the podocyte membrane) be transduced allowing the AFP widening response? (see below)

### A possible AFP stretch/flow transduction mechanism

Some of the mechanical stretch due to increased perfusion pressure will get exerted at the SD with the components being put under increased wall stress (hoop or paraxial stress due to the shape of the underlying capillary). Similarly, any increased filtrate flow due to increased perfusion pressure will have to pass through the SDs but exerting an increased radial stress on extracellular SD components pulling and stretching the SDD membranes. Thus, either increased wall stress or filtrate flow will both induce stretch deformations at the SD membranes.

While *in vitro* studies show that the identity of the podocyte mechanosensor remains unclear (114) one possible transduction pathway could involve stretch sensitive BK_Ca_ channels (115), which are bound to the SD protein complexes (both Nephrin and TRPC6. Figure 3A). Large conductance calcium activated potassium channels or BK_Ca_ channels could provide a mechanism with TRPC6 to fine-tune Ca^2+^ influx during normal glomerular function. The Slo1 subunits of BK_Ca_ bind to TRPC6 channels (116) (Figure 3A), which are expressed in the SD domain, cell body, and throughout the MP (117). The interactions between BK_Ca_ channels and actin filaments are complex and are likely to have multiple effects on the overall activity of BK_Ca_ channels (118). However, acute depolymerization of podocyte actin with cytochalasin-D did not affect BK_Ca_ channels (119), suggesting that any stretch activated response in AFP is not mediated by actin. While actin is thought of as being the mediator of protrusion and contraction, microtubules can also fulfill this function (118, 120).

How could differential responses to the same stretch stimulus arise in adjacent AFP and OFP, which share the same SD? This variability could be due to BK_Ca_ alone since the *KCNMA1* gene that codes for the 4 Slo1 subunits of BK_Ca_ has 35 exons, which can be alternatively spliced at 7 different sites. Thus, BK_Ca_ can have a multiplicity of variants sensitive to various pressure ranges but crucially, lots of different BK_Ca_ splice variants (and heteromers) can exist in a *single* cell (121–123). Its not unreasonable to hypothesize high stretch sensitivity splice variants/heteromers in AFP and low stretch sensitivity splice variants in OFP of the same cell and also on the other side of an SDD in an adjacent OFP. This could give the necessary asymmetric response in neighboring FPs. Highly stretch sensitive BK_Ca_ splice variants/heteromers should accordingly be located along AFP borders (SPS) and absent from OFP ones if this hypothesis works.

Crucially, in the rapid movement of AFP in response to pressure changes integrins can also alter their adhesive characteristics in response to cellular events via “inside-out” signaling (61) (IOS in Figure 3B), allowing them to take up the extra space on the GBM.

The alternative to a local stretch sensor is whole podocyte stretch sensing, with either BK_Ca_ or another mechanosensor. Here, the differential AFP/OFP mechanisms would rely on different AFP and OFP effector signaling pathways.

### Effacement, anchor widening and SPS loss

Widening of AFPs and the reduction of SPS under an elevated stimulus or a modified response could be a mechanism resulting in the production of effacement. This type of effacement appears to occur in many published electron micrographs [e.g., Figure 11 in (124)] and begs the question how much podocyte FP effacement is AFP widening in addition to OFP changes. Since SPS is a partially enclosed region, which accumulates/concentrates 10 kDa Dextran molecules but not molecules below 450 Da (111, 112) loss of this space points to a dysfunction in macromolecular transport by podocytes. Thus, effacement and proteinuria could fit, at least partly, with AFP widening and SPS loss in disease.

### AFP microtubule transport

Since AFPs are MPs (arising direct from cell bodies) its possible they have microtubules, podocyte MTs are oriented to convey cargoes both ways to and from the cell body along the MPs (16, 32). AFP associated microtubules might be organized for delivery to the periphery (i.e., GBM structural proteins from perinuclear Golgi apparatus) or delivery to the cell body (i.e., cargoes endocytosed from the GBM matrix) or both. The MPs that stretch away from the podocyte peri-nuclear region carrying many OFPs may transport in a different fashion to AFPs in SPS. The subtle distinctions between cytoplasmic transport domains in OFPs and AFPs might not be easy to determine.

## Conclusion

Clearly, any structural differences, defining characteristics and functional interactions surrounding AFPs and OFPs and any effects on SPS will need to be defined under normal *in vivo* podocyte conditions before sensible re-assessments of effacement or podocyte structural change can be made in disease.

## Conflict of Interest Statement

The author declares that the research was conducted in the absence of any commercial or financial relationships that could be construed as a potential conflict of interest.

## References

[1] Jarad G Cunningham J Shaw AS Miner JH. Proteinuria precedes podocyte abnormalities inLamb2-/- mice, implicating the glomerular basement membrane as an albumin barrier. J Clin Invest (2006) 116(8):2272–9. 10.1172/JCI28414 16886065 PMC1523402

[2] Satchell S. The role of the glomerular endothelium in albumin handling. Nat Rev Nephrol (2013) 9(12):717–25. 10.1038/nrneph.2013.197 24080802

[3] Wartiovaara J Ofverstedt LG Khoshnoodi J Zhang J Mäkelä E Sandin S Nephrin strands contribute to a porous slit diaphragm scaffold as revealed by electron tomography. J Clin Invest (2004) 114(10):1475–83. 10.1172/JCI22562C1 15545998 PMC525744

[4] Tryggvason K Wartiovaara J Molecular basis of glomerular permselectivity. Curr Opin Nephrol Hypertens (2001) 10(4):543–9 10.1097/00041552-200107000-00009 11458036

[5] Kerjaschki D Caught flat-footed: podocyte damage and the molecular bases of focal glomerulosclerosis. J Clin Invest (2001) 108(11):1583–7 10.1172/JCI200114629 11733553 PMC201002

[6] Kanwar YS Venkatachalam MA Ultrastructure of glomerulus and juxtaglomerular apparatus. In: Windhager EE, editor. Handbook of Physiology. New York, NY: Oxford University Press (1992). p. 3–40.

[7] Hall B Studies of normal glomerular structure by electron microscopy. 5th American Conference on Nephrotic Syndrome New York (1954).

[8] Deen WM Lazzara MJ Myers BD. Structural determinants of glomerular permeability. Am J Physiol Renal Physiol (2001) 281(4):F579–96. 11553505 10.1152/ajprenal.2001.281.4.F579

[9] Welsh GI Saleem MA. The podocyte cytoskeleton – key to a functioning glomerulus in health and disease. Nat Rev Nephrol (2012) 8(1):14–21. 10.1038/nrneph.2011.151 22025085

[10] Grahammer F Schell C Huber TB. The podocyte slit diaphragm – from a thin grey line to a complex signalling hub. Nat Rev Nephrol (2013) 9(10):587–98. 10.1038/nrneph.2013.169 23999399

[11] Weavers H Prieto-Sánchez S Grawe F Garcia-López A Artero R Wilsch-Bräuninger M The insect nephrocyte is a podocyte-like cell with a filtration slit diaphragm. Nature (2009) 457(7227):322–6. 10.1038/nature07526 18971929 PMC2687078

[12] Ichimura K Kurihara H Sakai T. Actin filament organization of foot processes in rat podocytes. J Histochem Cytochem (2003) 51(12):1589–600. 10.1177/002215540305101203 14623927

[13] Faul C Asanuma K Yanagida-Asanuma E Kim K Mundel P. Actin up: regulation of podocyte structure and function by components of the actin cytoskeleton. Trends Cell Biol (2007) 17(9):428–37. 10.1016/j.tcb.2007.06.006 17804239

[14] Cortes P Méndez M Riser BL Guérin CJ Rodríguez-Barbero A Hassett C F-actin fiber distribution in glomerular cells: structural and functional implications. Kidney Int (2000) 58(6):2452–61. 10.1046/j.1523-1755.2000.00428.x 11115078

[15] Farquhar MG Vernier RL Good RA. An electron microscope study of the glomerulus in nephrosis, glomerulonephritis, and lupus erythematosus. J Exp Med (1957) 106(5):649–60. 10.1084/jem.106.5.649 13475621 PMC2136823

[16] Kobayashi N Mundel P. A role of microtubules during the formation of cell processes in neuronal and non-neuronal cells. Cell Tissue Res (1998) 291(2):163–74. 10.1007/s004410050988 9426305

[17] Vasmant D Maurice M Feldmann G. Cytoskeleton ultrastructure of podocytes and glomerular endothelial cells in man and in the rat. Anat Rec (1984) 210(1):17–24. 10.1002/ar.1092100104 6541441

[18] Drenckhahn D Franke RP. Ultrastructural organization of contractile and cytoskeletal proteins in glomerular podocytes of chicken, rat, and man. Lab Invest (1988) 59(5):673–82. 3141719

[19] Kriz W Elger M Mundel P Lemley KV. Structure-stabilizing forces in the glomerular tuft. J Am Soc Nephrol (1995) 5(10):1731–9. 7787139 10.1681/ASN.V5101731

[20] Kriz W Mundel P Elger M The contractile apparatus of podocytes is arranged to counteract GBM expansion. Contrib Nephrol (1994) 107:1–9. 8004953 10.1159/000422954

[21] Endlich N Kress KR Reiser J Uttenweiler D Kriz W Mundel P Podocytes respond to mechanical stress in vitro. J Am Soc Nephrol (2001) 12(3):413–22. 11181788 10.1681/ASN.V123413

[22] Katoh K Kano Y Noda Y. Rho-associated kinase-dependent contraction of stress fibres and the organization of focal adhesions. J R Soc Interface (2011) 8(56):305–11. 10.1098/rsif.2010.0419 20826475 PMC3030825

[23] Wong JS Iorns E Rheault MN Ward TM Rashmi P Weber U Rescue of tropomyosin deficiency in *Drosophila* and human cancer cells by synaptopodin reveals a role of tropomyosin alpha in RhoA stabilization. EMBO J (2012) 31(4):1028–40. 10.1038/emboj.2011.464 22157816 PMC3280558

[24] Kawano Y Yoshimura T Kaibuchi K Smooth muscle contraction by small GTPase Rho. Nagoya J Med Sci (2002) 65(1–2):1–8. 12083286

[25] Stamenkovic I Skalli O Gabbiani G. Distribution of intermediate filament proteins in normal and diseased human glomeruli. Am J Pathol (1986) 125(3):465–75. 2432791 PMC1888470

[26] Yaoita E Franke WW Yamamoto T Kawasaki K Kihara I. Identification of renal podocytes in multiple species: higher vertebrates are vimentin positive/lower vertebrates are desmin positive. Histochem Cell Biol (1999) 111(2):107–15. 10.1007/s004180050340 10090571

[27] Yaoita E Wiche G Yamamoto T Kawasaki K Kihara I. Perinuclear distribution of plectin characterizes visceral epithelial cells of rat glomeruli. Am J Pathol (1996) 149(1):319–27. 8686756 PMC1865232

[28] Kurihara H Sunagawa N Kobayashi T Kimura K Takasu N Shike T. Monoclonal antibody P-31 recognizes a novel intermediate filament-associated protein (p250) in rat podocytes. Am J Physiol (1998) 274(5 Pt 2):F986–97. 9612338 10.1152/ajprenal.1998.274.5.F986

[29] Miner JH. Renal basement membrane components. Kidney Int (1999) 56(6):2016–24. 10.1046/j.1523-1755.1999.00785.x 10594777

[30] Cheng J Grassart A Drubin DG. Myosin 1E coordinates actin assembly and cargo trafficking during clathrin-mediated endocytosis. Mol Biol Cell (2012) 23(15):2891–904. 10.1091/mbc.E11-04-0383 22675027 PMC3408416

[31] Baas PW Black MM Banker GA. Changes in microtubule polarity orientation during the development of hippocampal neurons in culture. J Cell Biol (1989) 109(6 Pt 1):3085–94. 10.1083/jcb.109.6.3085 2592416 PMC2115969

[32] Kidd GJ Andrews SB Trapp BD. Organization of microtubules in myelinating Schwann cells. J Neurocytol (1994) 23(12):801–10. 10.1007/BF01268092 7897444

[33] Simons M Saffrich R Reiser J Mundel P. Directed membrane transport is involved in process formation in cultured podocytes. J Am Soc Nephrol (1999) 10(8):1633–9. 10446930 10.1681/ASN.V1081633

[34] Huber LA Pimplikar S Parton RG Virta H Zerial M Simons K. Rab8, a small GTPase involved in vesicular traffic between the TGN and the basolateral plasma membrane. J Cell Biol (1993) 123(1):35–45. 10.1083/jcb.123.1.35 8408203 PMC2119815

[35] Kim JH Konieczkowski M Mukherjee A Schechtman S Khan S Schelling JR Podocyte injury induces nuclear translocation of WTIP via microtubule-dependent transport. J Biol Chem (2010) 285(13):9995–10004. 10.1074/jbc.M109.061671 20086015 PMC2843245

[36] Guo JK Menke AL Gubler MC Clarke AR Harrison D Hammes A WT1 is a key regulator of podocyte function: reduced expression levels cause crescentic glomerulonephritis and mesangial sclerosis. Hum Mol Genet (2002) 11(6):651–9. 10.1093/hmg/11.6.651 11912180

[37] Kobayashi N Reiser J Schwarz K Sakai T Kriz W Mundel P. Process formation of podocytes: morphogenetic activity of microtubules and regulation by protein serine/threonine phosphatase PP2A. Histochem Cell Biol (2001) 115(3):255–66. 10.1007/s004180000242 11326753

[38] Noris MR Remuzzi G Non-muscle myosins and the podocyte. Clin Kidney J (2012) 5(2):94–101 10.1093/ckj/sfs032 PMC5783220 29497511

[39] Arif E Wagner MC Johnstone DB Wong HN George B Pruthi PA Motor protein Myo1c is a podocyte protein that facilitates the transport of slit diaphragm protein Neph1 to the podocyte membrane. Mol Cell Biol (2011) 31(10):2134–50. 10.1128/MCB.05051-11 21402783 PMC3133353

[40] Gbadegesin RA Winn MP Smoyer WE. Genetic testing in nephrotic syndrome – challenges and opportunities. Nat Rev Nephrol (2013) 9(3):179–84. 10.1038/nrneph.2012.286 23321566 PMC3702380

[41] Kerjaschki D. Polycation-induced dislocation of slit diaphragms and formation of cell junctions in rat kidney glomeruli: the effects of low temperature, divalent cations, colchicine, and cytochalasin B. Lab Invest (1978) 39(5):430–40. 104090

[42] Kriz W LeHir M. Pathways to nephron loss starting from glomerular diseases-insights from animal models. Kidney Int (2005) 67(2):404–19. 10.1111/j.1523-1755.2005.67097.x 15673288

[43] Faul C The Podocyte Cytoskeleton: Key to a Functioning Glomerulus in Health and Disease, in Podocytopathy (Contrib. Nephrol.). Basel: Karger (2014). p. 22–53.

[44] Miao J Fan Q Cui Q Zhang H Chen L Wang S Newly identified cytoskeletal components are associated with dynamic changes of podocyte foot processes. Nephrol Dial Transplant (2009) 24(11):3297–305. 10.1093/ndt/gfp338 19617259

[45] Li X Zhang X Li X Ding F Ding J. The role of survivin in podocyte injury induced by puromycin aminonucleoside. Int J Mol Sci (2014) 15(4):6657–73. 10.3390/ijms15046657 24747598 PMC4013653

[46] Liu Y Zhou QX Hou YY Lu B Yu C Chen J Actin polymerization-dependent increase in synaptic Arc/Arg3.1 expression in the amygdala is crucial for the expression of aversive memory associated with drug withdrawal. J Neurosci (2012) 32(35):12005–17. 10.1523/JNEUROSCI.0871-12.2012 22933785 PMC6621511

[47] Kurihara H Anderson JM Kerjaschki D Farquhar MG. The altered glomerular filtration slits seen in puromycin aminonucleoside nephrosis and protamine sulfate-treated rats contain the tight junction protein ZO-1. Am J Pathol (1992) 141(4):805–16. 1415478 PMC1886648

[48] Reiser J Kriz W Kretzler M Mundel P. The glomerular slit diaphragm is a modified adherens junction. J Am Soc Nephrol (2000) 11(1):1–8. 10616834 10.1681/ASN.V1111

[49] Shih NY Li J Karpitskii V Nguyen A Dustin ML Kanagawa O Congenital nephrotic syndrome in mice lacking CD2-associated protein. Science (1999) 286(5438):312–5. 10.1126/science.286.5438.312 10514378

[50] Huber TB Simons M Hartleben B Sernetz L Schmidts M Gundlach E Molecular basis of the functional podocin-nephrin complex: mutations in the NPHS2 gene disrupt nephrin targeting to lipid raft microdomains. Hum Mol Genet (2003) 12(24):3397–405. 10.1093/hmg/ddg360 14570703

[51] Lachapelle M Bendayan M. Contractile proteins in podocytes: immunocytochemical localization of actin and alpha-actinin in normal and nephrotic rat kidneys. Virchows Arch B Cell Pathol Incl Mol Pathol (1991) 60(2):105–11. 10.1007/BF02899534 1675506

[52] Kerjaschki D Sharkey DJ Farquhar MG. Identification and characterization of podocalyxin – the major sialoprotein of the renal glomerular epithelial cell. J Cell Biol (1984) 98(4):1591–6. 10.1083/jcb.98.4.1591 6371025 PMC2113206

[53] Huang TW Langlois JC. Podoendin. A new cell surface protein of the podocyte and endothelium. J Exp Med (1985) 162(1):245–67. 10.1084/jem.162.1.245 3891903 PMC2187700

[54] Sawada H Stukenbrok H Kerjaschki D Farquhar MG. Epithelial polyanion (podocalyxin) is found on the sides but not the soles of the foot processes of the glomerular epithelium. Am J Pathol (1986) 125(2):309–18. 3538890 PMC1888252

[55] Takeda T Go WY Orlando RA Farquhar MG. Expression of podocalyxin inhibits cell-cell adhesion and modifies junctional properties in Madin-Darby canine kidney cells. Mol Biol Cell (2000) 11(9):3219–32. 10.1091/mbc.11.9.3219 10982412 PMC14987

[56] Orlando RA Takeda T Zak B Schmieder S Benoit VM McQuistan T The glomerular epithelial cell anti-adhesin podocalyxin associates with the actin cytoskeleton through interactions with ezrin. J Am Soc Nephrol (2001) 12(8):1589–98. 11461930 10.1681/ASN.V1281589

[57] Takeda T McQuistan T Orlando RA Farquhar MG. Loss of glomerular foot processes is associated with uncoupling of podocalyxin from the actin cytoskeleton. J Clin Invest (2001) 108(2):289–301. 10.1172/JCI200112539 11457882 PMC203027

[58] Doyonnas R Kershaw DB Duhme C Merkens H Chelliah S Graf T Anuria, omphalocele, and perinatal lethality in mice lacking the CD34-related protein podocalyxin. J Exp Med (2001) 194(1):13–27. 10.1084/jem.194.1.13 11435469 PMC2193439

[59] Patrie KM Drescher AJ Goyal M Wiggins RC Margolis B. The membrane-associated guanylate kinase protein MAGI-1 binds megalin and is present in glomerular podocytes. J Am Soc Nephrol (2001) 12(4):667–77. 11274227 10.1681/ASN.V124667

[60] Patrie KM Drescher AJ Welihinda A Mundel P Margolis B. Interaction of two actin-binding proteins, synaptopodin and alpha-actinin-4, with the tight junction protein MAGI-1. J Biol Chem (2002) 277(33):30183–90. 10.1074/jbc.M203072200 12042308

[61] Blattner SM Kretzler M. Integrin-linked kinase in renal disease: connecting cell-matrix interaction to the cytoskeleton. Curr Opin Nephrol Hypertens (2005) 14(4):404–10. 10.1097/01.mnh.0000172730.67746.5b 15931012

[62] Goode NP Shires M Khan TN Mooney AF. Expression of alpha-actinin-4 in acquired human nephrotic syndrome: a quantitative immunoelectron microscopy study. Nephrol Dial Transplant (2004) 19(4):844–51. 10.1093/ndt/gfg620 15031339

[63] Dai C Stolz DB Bastacky SI St-Arnaud R Wu C Dedhar S Essential role of integrin-linked kinase in podocyte biology: bridging the integrin and slit diaphragm signaling. J Am Soc Nephrol (2006) 17(8):2164–75. 10.1681/ASN.2006010033 16837631

[64] Schmieder S Nagai M Orlando RA Takeda T Farquhar MG. Podocalyxin activates RhoA and induces actin reorganization through NHERF1 and Ezrin in MDCK cells. J Am Soc Nephrol (2004) 15(9):2289–98. 10.1097/01.ASN.0000135968.49899.E8 15339978

[65] Wiggins RC. The spectrum of podocytopathies: a unifying view of glomerular diseases. Kidney Int (2007) 71(12):1205–14. 10.1038/sj.ki.5002222 17410103

[66] Kriz W. Podocyte is the major culprit accounting for the progression of chronic renal disease. Microsc Res Tech (2002) 57(4):189–95. 10.1002/jemt.10072 12012382

[67] Wharram BL Goyal M Wiggins JE Sanden SK Hussain S Filipiak WE Podocyte depletion causes glomerulosclerosis: diphtheria toxin-induced podocyte depletion in rats expressing human diphtheria toxin receptor transgene. J Am Soc Nephrol (2005) 16(10):2941–52. 10.1681/ASN.2005010055 16107576

[68] Sachs N Sonnenberg A. Cell-matrix adhesion of podocytes in physiology and disease. Nat Rev Nephrol (2013) 9(4):200–10. 10.1038/nrneph.2012.291 23338211

[69] Petermann AT Pippin J Krofft R Blonski M Griffin S Durvasula R Viable podocytes detach in experimental diabetic nephropathy: potential mechanism underlying glomerulosclerosis. Nephron Exp Nephrol (2004) 98(4):e114–23. 10.1159/000081555 15627794

[70] Verzola D Gandolfo MT Ferrario F Rastaldi MP Villaggio B Gianiorio F Apoptosis in the kidneys of patients with type II diabetic nephropathy. Kidney Int (2007) 72(10):1262–72. 10.1038/sj.ki.5002531 17851466

[71] Meyer TW Bennett PH Nelson RG. Podocyte number predicts long-term urinary albumin excretion in Pima Indians with type II diabetes and microalbuminuria. Diabetologia (1999) 42(11):1341–4. 10.1007/s001250051447 10550418

[72] Pagtalunan ME Miller PL Jumping-Eagle S Nelson RG Myers BD Rennke HG Podocyte loss and progressive glomerular injury in type II diabetes. J Clin Invest (1997) 99(2):342–8. 10.1172/JCI119163 9006003 PMC507802

[73] Steffes MW Schmidt D McCrery R Basgen JM International Diabetic Nephropathy Study Group. Glomerular cell number in normal subjects and in type 1 diabetic patients. Kidney Int (2001) 59(6):2104–13. 10.1046/j.1523-1755.2001.0590062104.x 11380812

[74] White KE Bilous RW Marshall SM El Nahas M Remuzzi G Piras G Podocyte number in normotensive type 1 diabetic patients with albuminuria. Diabetes (2002) 51(10):3083–9. 10.2337/diabetes.51.10.3083 12351451

[75] Griffin SV Petermann AT Durvasula RV Shankland SJ. Podocyte proliferation and differentiation in glomerular disease: role of cell-cycle regulatory proteins. Nephrol Dial Transplant (2003) 18(Suppl 6):vi8–13. 10.1093/ndt/gfg1069 12953035

[76] Sugimoto H Mundel TM Sund M Xie L Cosgrove D Kalluri R. Bone-marrow-derived stem cells repair basement membrane collagen defects and reverse genetic kidney disease. Proc Natl Acad Sci U S A (2006) 103(19):7321–6. 10.1073/pnas.0601436103 16648256 PMC1464339

[77] Prodromidi EI Poulsom R Jeffery R Roufosse CA Pollard PJ Pusey CD Bone marrow-derived cells contribute to podocyte regeneration and amelioration of renal disease in a mouse model of Alport syndrome. Stem Cells (2006) 24(11):2448–55. 10.1634/stemcells.2006-0201 16873763

[78] Ronconi E Sagrinati C Angelotti ML Lazzeri E Mazzinghi B Ballerini L Regeneration of glomerular podocytes by human renal progenitors. J Am Soc Nephrol (2009) 20(2):322–32. 10.1681/ASN.2008070709 19092120 PMC2637058

[79] Romagnani P Parietal epithelial cells: their role in health and disease. Contrib Nephrol (2011) 169:23–36 10.1159/000313943 21252509

[80] Schulte K Berger K Boor P Jirak P Gelman IH Arkill KP Origin of parietal podocytes in atubular glomeruli mapped by lineage tracing. J Am Soc Nephrol (2014) 25(1):129–41. 10.1681/ASN.2013040376 24071005 PMC3871778

[81] Hackl MJ Burford JL Villanueva K Lam L Suszták K Schermer B Tracking the fate of glomerular epithelial cells in vivo using serial multiphoton imaging in new mouse models with fluorescent lineage tags. Nat Med (2013) 19(12):1661–6. 10.1038/nm.3405 24270544 PMC3884556

[82] Wiggins JE Goyal M Sanden SK Wharram BL Shedden KA Misek DE Podocyte hypertrophy, “adaptation,” and “decompensation” associated with glomerular enlargement and glomerulosclerosis in the aging rat: prevention by calorie restriction. J Am Soc Nephrol (2005) 16(10):2953–66. 10.1681/ASN.2005050488 16120818

[83] Kriz W Kretzler M Nagata M Provoost AP Shirato I Uiker S A frequent pathway to glomerulosclerosis: deterioration of tuft architecture-podocyte damage-segmental sclerosis. Kidney Blood Press Res (1996) 19(5):245–53. 10.1159/000174083 8956236

[84] Lohmann F Sachs M Meyer TN Sievert H Lindenmeyer MT Wiech T UCH-L1 induces podocyte hypertrophy in membranous nephropathy by protein accumulation. Biochim Biophys Acta (2014) 1842(7):945–58. 10.1016/j.bbadis.2014.02.011 24583340

[85] Rüster C Bondeva T Franke S Förster M Wolf G. Advanced glycation end-products induce cell cycle arrest and hypertrophy in podocytes. Nephrol Dial Transplant (2008) 23(7):2179–91. 10.1093/ndt/gfn085 18344241

[86] Wolf G Schroeder R Thaiss F Ziyadeh FN Helmchen U Stahl RA. Glomerular expression of p27Kip1 in diabetic db/db mouse: role of hyperglycemia. Kidney Int (1998) 53(4):869–79. 10.1111/j.1523-1755.1998.00829.x 9551393

[87] Shankland SJ Floege J Thomas SE Nangaku M Hugo C Pippin J Cyclin kinase inhibitors are increased during experimental membranous nephropathy: potential role in limiting glomerular epithelial cell proliferation in vivo. Kidney Int (1997) 52(2):404–13. 10.1038/ki.1997.347 9263996

[88] Petermann AT Pippin J Durvasula R Pichler R Hiromura K Monkawa T Mechanical stretch induces podocyte hypertrophy in vitro. Kidney Int (2005) 67(1):157–66. 10.1111/j.1523-1755.2005.00066.x 15610239

[89] Gödel M Hartleben B Herbach N Liu S Zschiedrich S Lu S Role of mTOR in podocyte function and diabetic nephropathy in humans and mice. J Clin Invest (2011) 121(6):2197–209. 10.1172/JCI44774 21606591 PMC3104746

[90] Kriz W Shirato I Nagata M LeHir M Lemley KV. The podocyte’s response to stress: the enigma of foot process effacement. Am J Physiol Renal Physiol (2013) 304(4):F333–47. 10.1152/ajprenal.00478.2012 23235479

[91] Shirato I Sakai T Kimura K Tomino Y Kriz W. Cytoskeletal changes in podocytes associated with foot process effacement in Masugi nephritis. Am J Pathol (1996) 148(4):1283–96. 8644869 PMC1861509

[92] Kretzler M Koeppen-Hagemann I Kriz W. Podocyte damage is a critical step in the development of glomerulosclerosis in the uninephrectomised-desoxycorticosterone hypertensive rat. Virchows Arch (1994) 425(2):181–93. 10.1007/BF00230355 7952502

[93] Topham PS Haydar SA Kuphal R Lightfoot JD Salant DJ. Complement-mediated injury reversibly disrupts glomerular epithelial cell actin microfilaments and focal adhesions. Kidney Int (1999) 55(5):1763–75. 10.1046/j.1523-1755.1999.00407.x 10231439

[94] Whiteside CI Cameron R Munk S Levy J. Podocytic cytoskeletal disaggregation and basement-membrane detachment in puromycin aminonucleoside nephrosis. Am J Pathol (1993) 142(5):1641–53. 8494056 PMC1886918

[95] Smoyer WE Mundel P Gupta A Welsh MJ. Podocyte alpha-actinin induction precedes foot process effacement in experimental nephrotic syndrome. Am J Physiol (1997) 273(1):F150–7. 9249603 10.1152/ajprenal.1997.273.1.F150

[96] Michaud JL Lemieux LI Dubé M Vanderhyden BC Robertson SJ Kennedy CR. Focal and segmental glomerulosclerosis in mice with podocyte-specific expression of mutant alpha-actinin-4. J Am Soc Nephrol (2003) 14(5):1200–11. 10.1097/01.ASN.0000059864.88610.5E 12707390

[97] Kaplan JM Kim SH North KN Rennke H Correia LA Tong HQ Mutations in ACTN4, encoding alpha-actinin-4, cause familial focal segmental glomerulosclerosis. Nat Genet (2000) 24(3):251–6. 10.1038/73456 10700177

[98] Asanuma K Mundel P. The role of podocytes in glomerular pathobiology. Clin Exp Nephrol (2003) 7(4):255–9. 10.1007/s10157-003-0259-6 14712353

[99] Rood IM Deegens JK Wetzels JF. Genetic causes of focal segmental glomerulosclerosis: implications for clinical practice. Nephrol Dial Transplant (2012) 27(3):882–90. 10.1093/ndt/gfr771 22334613

[100] Yuan H Takeuchi E Taylor GA McLaughlin M Brown D Salant DJ. Nephrin dissociates from actin, and its expression is reduced in early experimental membranous nephropathy. J Am Soc Nephrol (2002) 13(4):946–56. 11912254 10.1681/ASN.V134946

[101] Liu Y Yerushalmi GM Grigera PR Parsons JT. Mislocalization or reduced expression of Arf GTPase-activating protein ASAP1 inhibits cell spreading and migration by influencing Arf1 GTPase cycling. J Biol Chem (2005) 280(10):8884–92. 10.1074/jbc.M412200200 15632162

[102] Zhu J Sun N Aoudjit L Li H Kawachi H Lemay S Nephrin mediates actin reorganization via phosphoinositide 3-kinase in podocytes. Kidney Int (2008) 73(5):556–66. 10.1038/sj.ki.5002691 18033240

[103] Asanuma K Yanagida-Asanuma E Faul C Tomino Y Kim K Mundel P. Synaptopodin orchestrates actin organization and cell motility via regulation of RhoA signalling. Nat Cell Biol (2006) 8(5):485–91. 10.1038/ncb1400 16622418

[104] Kistler AD Altintas MM Reiser J. Podocyte GTPases regulate kidney filter dynamics. Kidney Int (2012) 81(11):1053–5. 10.1038/ki.2012.12 22584591 PMC3354621

[105] Yoo TH Pedigo CE Guzman J Correa-Medina M Wei C Villarreal R Sphingomyelinase-like phosphodiesterase 3b expression levels determine podocyte injury phenotypes in glomerular disease. J Am Soc Nephrol (2014) 26(1):133–47. 10.1681/ASN.2013111213 24925721 PMC4279736

[106] Gautier A Bernhard W Oberling C [The existence of a pericapillary lacunar apparatus in the malpighian glomeruli revealed by electronic microscopy]. C R Seances Soc Biol Fil (1950) 144(23–24):1605–7. 14822344

[107] Elias H Allara E Elias PM Murthy AS The podocytes, re-examined. Z Mikrosk Anat Forsch (1965) 72(2):344–65. 5826826

[108] Neal CR Crook H Bell E Harper SJ Bates DO. Three-dimensional reconstruction of glomeruli by electron microscopy reveals a distinct restrictive urinary subpodocyte space. J Am Soc Nephrol (2005) 16(5):1223–35. 10.1681/ASN.2004100822 15829713

[109] Neal CR Muston PR Njegovan D Verrill R Harper SJ Deen WM Glomerular filtration into the subpodocyte space is highly restricted under physiological perfusion conditions. Am J Physiol Renal Physiol (2007) 293(6):F1787–98. 10.1152/ajprenal.00157.2007 17715264

[110] Arkill KP Qvortrup K Starborg T Mantell JM Knupp C Michel CC Resolution of the three dimensional structure of components of the glomerular filtration barrier. BMC Nephrol (2014) 15:24. 10.1186/1471-2369-15-24 24484633 PMC3922634

[111] Salmon AH Toma I Sipos A Muston PR Harper SJ Bates DO Evidence for restriction of fluid and solute movement across the glomerular capillary wall by the subpodocyte space. Am J Physiol Renal Physiol (2007) 293(6):F1777–86. 10.1152/ajprenal.00187.2007 17804486

[112] Kistler AD Caicedo A Abdulreda MH Faul C Kerjaschki D Berggren PO In vivo imaging of kidney glomeruli transplanted into the anterior chamber of the mouse eye. Sci Rep (2014) 4:3872. 10.1038/srep03872 24464028 PMC3902446

[113] Thomson SC Blantz RC. Biophysics of glomerular filtration. Compr Physiol (2012) 2(3):1671–99. 10.1002/cphy.c100089 23723020

[114] Endlich N Endlich K. The challenge and response of podocytes to glomerular hypertension. Semin Nephrol (2012) 32(4):327–41. 10.1016/j.semnephrol.2012.06.004 22958487

[115] Morton MJ Hutchinson K Mathieson PW Witherden IR Saleem MA Hunter M. Human podocytes possess a stretch-sensitive, Ca2+-activated K+ channel: potential implications for the control of glomerular filtration. J Am Soc Nephrol (2004) 15(12):2981–7. 10.1097/01.ASN.0000145046.24268.0D 15579500

[116] Kim EY Alvarez-Baron CP Dryer SE. Canonical transient receptor potential channel (TRPC)3 and TRPC6 associate with large-conductance Ca2+-activated K+ (BKCa) channels: role in BKCa trafficking to the surface of cultured podocytes. Mol Pharmacol (2009) 75(3):466–77. 10.1124/mol.108.051912 19052171 PMC2645922

[117] Reiser J Polu KR Möller CC Kenlan P Altintas MM Wei C TRPC6 is a glomerular slit diaphragm-associated channel required for normal renal function. Nat Genet (2005) 37(7):739–44. 10.1038/ng1592 15924139 PMC1360984

[118] Dryer SE Reiser J. TRPC6 channels and their binding partners in podocytes: role in glomerular filtration and pathophysiology. Am J Physiol Renal Physiol (2010) 299(4):F689–701. 10.1152/ajprenal.00298.2010 20685822 PMC2957253

[119] Kim EY Suh JM Chiu YH Dryer SE. Regulation of podocyte BK(Ca) channels by synaptopodin, Rho, and actin microfilaments. Am J Physiol Renal Physiol (2010) 299(3):F594–604. 10.1152/ajprenal.00206.2010 20630939 PMC2944294

[120] Etienne-Manneville S. Actin and microtubules in cell motility: which one is in control? Traffic (2004) 5(7):470–7. 10.1111/j.1600-0854.2004.00196.x 15180824

[121] Kim EY Choi KJ Dryer SE. Nephrin binds to the COOH terminus of a large-conductance Ca2+-activated K+ channel isoform and regulates its expression on the cell surface. Am J Physiol Renal Physiol (2008) 295(1):F235–46. 10.1152/ajprenal.00140.2008 18480178 PMC2494500

[122] Kim EY Ridgway LD Zou S Chiu YH Dryer SE. Alternatively spliced C-terminal domains regulate the surface expression of large conductance calcium-activated potassium channels. Neuroscience (2007) 146(4):1652–61. 10.1016/j.neuroscience.2007.03.038 17478049 PMC1995407

[123] Beisel KW Rocha-Sanchez SM Ziegenbein SJ Morris KA Kai C Kawai J Diversity of Ca2+-activated K+ channel transcripts in inner ear hair cells. Gene (2007) 386(1–2):11–23. 10.1016/j.gene.2006.07.023 17097837

[124] Pavenstadt H Kriz W Kretzler M Cell biology of the glomerular podocyte. Physiol Rev (2003) 83(1):253–307 10.1152/physrev.00020.2002 12506131

